# Reframing physically active learning as movement-centred pedagogy: a European priority action framework

**DOI:** 10.1186/s12966-023-01503-4

**Published:** 2023-08-25

**Authors:** Anna Chalkley, Mathias Brekke Mandelid, Amika Singh, Geir Kare Resaland, Andrew Daly-Smith

**Affiliations:** 1 Centre for Physically Active Learning, Faculty of Education, Arts and Sports, Western Norway University of Applied Sciences, Bergen, Norway; 2 Centre for Applied Education Research, Wolfson Centre for Applied Health Research Bradford Royal Infirmary, Bradford, West Yorkshire UK; 3 Faculty of Humanities, Sports and Educational Science, Department of Sports, Physical Education and Outdoor Studies, University of South-Eastern Norway, Bø, Norway; 4 Mulier Institute Utrecht, Utrecht, The Netherlands; 5 Faculty of Health Studies, University of Bradford, Bradford, UK

**Keywords:** Physically active learning, School-based physical activity, Policy, Co-development, Teachers, Movement-centred pedagogy

## Abstract

**Background:**

Physically active learning (PAL) has emerged as a promising way of eliciting health and education-based outcomes for pupils. Concurrently, research suggests large variability in how PAL is perceived, operationalized, and prioritized in practice across Europe. Therefore, this study aimed to co-develop a framework for action to support the adoption and implementation of PAL.

**Methods:**

Adopting a design thinking approach, 40 international stakeholders representing 13 countries engaged in an idea generation workshop during a two-day PAL international conference. Participants included professionals from research (n = 20), practice (n = 4) and policy (n = 1) or a combination (n = 15). Their experience with PAL ranged from none to 19 years (with an average of 3.9 years). Participants were allocated into one of six heterogeneous and multidisciplinary groups and led through interactive tasks to identify: the landscape for PAL across Europe, barriers to the adoption and implementation of PAL, and key objectives for research, policy and practice to improve the adoption and implementation of PAL. All discussions were audio recorded and prioritized objectives were transcribed verbatim and analysed using inductive qualitative content analysis.

**Results:**

Five interlinked and mutually reinforcing themes were identified: (1) Integration of the health and education paradigms (2) Coherent national policy and decision making (3) Building confident and competent teachers (4) Adopting a whole school approach for PAL (5) Strengthening the evidence base for PAL.

**Conclusions:**

The priority action framework identifies five key areas for action to facilitate PAL adoption and implementation across Europe. Central to the success of border uptake of PAL is the integration of the health and education paradigms. To achieve this aim, reframing PAL as movement-centered pedagogy would provide a more holistic and inclusive perspective.

**Supplementary Information:**

The online version contains supplementary material available at 10.1186/s12966-023-01503-4.

## Background

One component of a whole school approach which has received growing interest in recent years is physically active learning (PAL). Described as the integration of movement within delivery of academic content [1], PAL has emerged as a low-cost/no-cost way to reduce sitting time and increase physical activity behaviour during teaching without competing for curriculum learning time [2]. Moreover, there is a growing evidence base surrounding the small but positive effects on cognition and academic performance [3–5]. Thus, PAL could reap reciprocal benefits to children’s education and health, if implemented in schools.

Despite its promise, the adoption and implementation of PAL has been slow [6] suggesting a gap between effective practices and policies to promote physical activity and what happens in the day-to-day realities of schools. Indeed, research has demonstrated that there are multiple levels of influence on, and variability in, how PAL is perceived, operationalized and prioritized in practice across Europe which would account for some of this incongruity. Chalkley et al. (2022) highlighted how a country’s national policies influenced school priorities and teachers’ professional practices in relation to PAL [7]. This demonstrates the need for engagement of a wide range of stakeholders working with(in) education settings so that the knowledge, insights, and experiences of those who are either involved in or potentially affected by the implementation of policies and interventions are valued and prioritized. Movements such as Creating Active Schools in the UK highlight the importance of aligning education and health policy, practice and research, to facilitate a culture shift at all levels of the school system. To achieve this, trans disciplinarity is required to integrate the traditional health disciplines of health promotion, medicine, epidemiology and psychology with the educational disciplines of pedagogy and learning theory [8]. As a result, a conceptual shift towards strategies or practices which serve to cross and/or connect the health education nexus can be observed [9]. Their success, however, is dependent on the political will to implement and support them [10].

Previous research has highlighted behaviours that prompt individual teachers to adopt and implement PAL [11], however there is a need to address wider system level factors and scalable actions to support the adoption and implementation of PAL if its potential in providing positive outcomes for pupils is to realised. The aim of the current study was to co-develop a framework for action to support the adoption and implementation of PAL across Europe. In doing so we sought consensus on priorities for action across research, policy and practice, perceived to have the biggest potential impact for change at a national level.

## Methods

### Overview

The co-development process took place during a two-day international PAL congress in April 2022 in Zwolle, the Netherlands. The event was organized as a multiplier event for the ACTivate project^1^ and advertised through the ACTivate consortium countries’ PAL networks, and via social media. Registered delegates were notified of the intention of the workshop prior to the congress and invited to participate in the study by email. During the congress registration, and prior to start of the workshop, delegates were reminded that discussions would be recorded and used for data collection. Delegates were also informed that participating in the research was optional and would not affect their ability to participate in the workshop. All delegates (n = 40) provided their consent.

The ACTivate congress was targeted at anyone with an interest in PAL and school-based physical activity and included an audience consisting of researchers, policy makers and teacher educators. The conference program consisted of two parallel streams, the first was aimed at researchers, policy makers and teacher educators (held in English), the second at classroom/PE teachers (held in Dutch). The workshop was held as part of the first stream and open to anyone who felt confident to converse in English, consequently, the participant sample included 40 researchers, policy makers and teacher educators from 13 countries (12 European and one South American). Twenty-five participants registered with a singular role; 20 researchers, one policy maker and four teacher educators. Fifteen participants self-identified as having a hybrid role, that is, one which combined one or more of the three professional groups. Of these, 10 participants reported roles which combined research and teacher education and five reported roles which combined research, policy and teacher education. Ten participants possessed a teaching qualification (for example, a postgraduate certificate in education, a Masters in Education or a diploma in education and training). Participants’ self-identified experience of working with PAL varied and ranged from no experience to 19 years, with an average of 3.9 years, see Table 1 for a summary of participant characteristics.

**Table 1:** Summary of participant characteristics

Stakeholder Group	Typical role	Countries represented	Time in current role (years, mean (range))	PAL based experience (years, mean(range))
Researcher (n = 20)	Research assistant, PhD student, Associate professor	Brazil, UK, Italy, Germany, Estonia, Portugal, Denmark, The Netherlands	14.9 (1–20)	9.3 (1–10)
Policy maker (n = 1)	Policy advisor	The Netherlands	2	0
Teacher Educator (n = 4)	Teacher trainer, educational didactician	Belgium, Spain, Portugal, UK	13.2 (2–20)	3 (0–6)
Hybrid – Research and Teacher Education (n = 10)	Researcher and teacher trainer	Norway, Spain, UK, Estonia, Finland	6.1 (2–20)	2.1 (0–5)
Hybrid – Research, Policy and Teacher Education (n = 5)	Knowledge consultant, Project manager, Insight manager	The Netherlands, Denmark	4.8 (4–8)	6.6 (1–19)

### Data collection

Participants took part in a facilitated idea generation workshop which drew on design thinking philosophy [12] to encourage multi-stakeholder discussion, critical reflection and ideation using a solution focused approach. Design thinking is aligned with participatory research methods and is intended to generate ‘bottom-up’ and well as ‘top-down’ solutions which complement national contexts and are thus potentially more effective and sustainable [13].

Participants were organized into six heterogeneous and multidisciplinary groups based on demographic information submitted during recruitment. Attention was given to ensure diversity in each group in terms of role, length of experience of working with PAL and countries represented. Every group was supported by a core member of the ACTivate project team who encouraged participants to articulate, and elaborate on, their thoughts. Concurrent discussions during the workshop were audio recorded using dictaphones placed on each table.

The first author,trained in participatory methods, facilitated the workshop and engaged participants in a series of interactive tasks. The first served as an ice breaker where participants placed themselves on a virtual continuum according to their perception of the available infrastructure for PAL in their country, before returning to their groups to introduce themselves, their backgrounds and their placement on the continuum. A definition of PAL, “the integration of movement within the delivery of academic content” [1] was provided to foster a shared understanding in each group and facilitate discussion of the PAL landscape in their respective country in terms of its status in relation to research, policy, and practice.

For the second task, participants were provided with post-it notes to answer the first question, “What are some of the challenges or barriers your country faces to improving the adoption and implementation of PAL?” Individuals were encouraged to share, discuss and capture their tacit knowledge on the post-it notes. These were subsequently presented for reviewing by the other groups during a period of sharing and reflection. Subsequentially, groups were invited to add to their post-it notes before being asked to address the second task question, “What are some of the key objectives for research, policy and practice to improving the adoption and implementation of PAL?” In responding, groups were asked to write their key objectives for research, policy and practice on postcards before ranking them from highest to lowest priority. Once again, each group was encouraged to review the objectives of the other groups, before subsequently adding to their original list and reprioritizing if appropriate. The workshop lasted for two hours and thirty minutes.

### Data analysis

Group discussions, post-it notes, and prioritized objectives were transcribed verbatim into Microsoft Word. Data were analysed by the first author using qualitative content analysis with the aid of Nvivo (QSR 1.6.1) to synthesise data into categories based on inference and interpretation [14]. Using an inductive approach and an open coding frame to allow broad concepts and patterns to be identified, data were coded into themes based on meanings and impressions at both the latent and semantic level [15]. Interpretations of the post-it notes and priorities for action were supported by participants’ quotes. Thematic codes were grouped into larger categories or subthemes and subsequently main themes. An audit trail was maintained to provide transparency in the decision-making process and contribute to rigor of the analysis. Candidate themes were presented to the co-authors and interpretations were openly discussed and challenged by critically probing for explanations to achieve a final consensus.

## Results

Thirty-four unique priorities for PAL were identified across the six multi-stakeholder groups (17 policy related, 21 practice related and 25 research related). Although groups were asked to prioritise actions, many referred to the need to take a whole systems approach to the development of PAL to reflect the need to galvanise and reinforce actions by all stakeholders. However, whilst components of a whole-systems approach were identified, understanding of how these components could or should interact was not articulated. Therefore, what is presented serves as the basis of a framework for action to support the adoption and implementation of PAL across Europe. The five identified themes were: (1) Integration of the health and education paradigms (2) Coherent national policy and decision making (3) Building confident and competent teachers (4) Adopting a whole school approach for PAL (5) Strengthening the evidence base for PAL. Collectively, the themes address the structural determinants of the adoption and implementation of PAL, which operate at multiple system levels. Each shall be explored in further detail below.

### Integration of the health and education paradigms

During discussions around barriers to the adoption and implementation of PAL, participants suggested that a contributing factor could be due to the tensions some may experience when navigating between the dominant paradigms of PAL, that of health and education. There was a consensus among participants that much of the research relating to PAL had emerged from a preventative public health perspective with a “top-down” focus on minutes of physical activity during curricular time as the main outcome. This emphasis on PAL as a physical activity intervention, was perceived by many as unhelpful when trying to convey the relevance of PAL to teachers:

*Health education is always present, but they don’t connect with physical activity or physical activities, outside school, or physically active learning. So maybe the educational approach, maybe it’s better* (Group 3, P4, female, Portugal).

Some argued that the dominance of the health paradigm associated with PAL has meant that: *“teachers relate PAL to very high intensity physical activity”* (Group 6, P2 male, Norway) and often perceive it as synonymous with Physical Education (PE) and/or needing to be trained in PE to be able to use movement as part of their teaching. However, participants felt that it would be more helpful to reframe physical activity as a by-product of PAL, as opposed to the main goal, to assist teachers in understanding and exploring the use of PAL from a more holistic perspective. By focusing on the meaning of the movement within the teacher’s pedagogical practice, participants reflected that it would strengthen the link between PAL and the purpose of education, thus allowing the quality of the movement to be emphasized rather than quantity of movement:

*We talked about the perceiving PAL as a learning mode or as a tool in your toolbox as a teacher, not as a specific task that you have to fulfil so it’s something I choose because it helps my teaching. It helps the process, the learning process, or the purpose I have, instead of seeing PAL as an individual purpose; besides teaching I have to do PAL* (Group 4, P5, male, Denmark).

Such confluence of the two paradigms wouldn’t be to privilege one over the other but rather to acknowledge and value their complementarity by defining and articulating the shared agenda more clearly.

*So, this [PAL] is the actually the cross issue. I think the cross section is active learning, so, we should present it in this manner. But we should say that this is actually originally addressing educational field but we will also add a little part to solving the public health problem* (Group 1, P5, female, Estonia).

Given the dominance of more ‘bottom-up’ and practice-led approaches to PAL, which were shared, participants identified an important priority as increasing awareness of PAL and addressing some of the ambiguity surrounding what PAL is and what it looks like in practice. For example, participants shared the use of informal professional learning communities where teachers could engage in reflective practice and resources and ideas for PAL across different curriculum subjects could be shared, tested, and refined. One important consideration was, therefore, the type of messaging that could be used in encouraging teachers to adopt PAL. Participants suggested that it would be important to include both educational and health but warned that attention should be given to using terminology and language which would resonate with teachers:

*When we are doing things in practice with the teacher, we need to talk in their keywords. Yeah, so listen at that time to the meaning, because that’s where the work starts, finding the meaning*. (Group 3, P1, female, Denmark)

### Coherent national policy and decision making

Policy related actions were consistently identified by participants as having the greatest potential to help prioritise actions and facilitate the adoption and implementation of PAL on a wide scale. Participants reflected that those countries where the use of PAL was perceived to be more established and advanced were ones which had embraced PAL from a more holistic perspective rather than one which compartmentalises teaching and learning in the way that education is practiced and governed. That is, one where responsibility for the nurturing of educational outcomes is divided between different subjects or curriculum areas. For example, Norway was a country which was consistently referred to by participants as an exemplar for how PAL was supported within the wider system, as one participant commented: *“If I was a teacher in Norway, it would be easier to do PAL”* (G2, P 7, female, Netherlands). This was because the system was believed to allow curriculum practices, such as PAL, which encourage pupils to engage in meaning-making processes through their connection with, and interpretation of, the topic via movement.

*In Norway, we have the curriculum, a new curriculum which allows or gives teachers opportunities to be more flexible in their teaching. So the aims are a little bit broader. So we see that PAL can be something they can use to work on those aims and be useful* (G6, P2, male, Norway).

However, participants emphasised the complexity associated with engaging the wide range of stakeholders linked with school-based practice and the interplay between different organisational layers inherent within the educational system:

*We show like this is the chain, the school system chain. So you have policy, national, local policy. You have municipality, that are the ones that take the policy and make it happen in the municipality. You have the school leaders, the teachers, your pupils, and just to illustrate that it’s a chain* (P4, Group 4 – male, Denmark).

Although bottom-up approaches were emphasised as important to promoting a more integrated discourse (e.g., the development of different curriculum practices as part of a professional learning community), top-down approaches were perceived as necessary to challenge educational norms and empower teachers by providing them with the confidence and competence to be able to use PAL in their everyday practice. For example, one recommendation was the introduction of the assessment of PAL as a mandatory part of school inspections. Participants thought this would help to provide a mechanism of accountability, and if supported by clear guidance, ownership of PAL by the schools and trust among teachers. Importantly, participants argued that bottom-up and top-down approaches were not mutually exclusive and added that it was necessary for policy to be co-produced, that is for policy makers to draw on the expertise of teachers, teacher educators and other stakeholders to ensure alignment to an agreed paradigm. This would serve to prevent disjointed decision making and avoid any policy actions which could operate in conflict with practice or inhibit stakeholders’ ability to implement the policy. This was experienced by one participant from Denmark:

*PAL is actually not a mandatory part of our teacher education. So, we are saying in school, they have to do it, but we’re not teaching them how to do it. They can choose it, or they can choose not to learn it, which is crazy when you think about it. But the gap here is actually on a political level, because it’s two different ministries. So you have a Ministry of Teaching, and you have a Ministry of Education, which is crazy* (Group 3, P1, Female, Denmark).

In addition to practice being reflected in the decision-making process, participants also called for stronger links between research and policy, for example by conducting policy relevant research which has societal value, so that the implications for policy makers are clear and any recommendations would be more likely to be considered:

*I think because at least still in Denmark, when they make policies, it’s not the teachers that are asked, it’s the researchers. And there the mechanisms are important for them to argue how much this should be a part of a law* (Group 4, male, Denmark).

Consequently, participants believed that replication of such a system would require strategic advocacy and support not only from multisector stakeholders across policy and practice but also those from all levels of the system, as one participant noted:

*We have to know who we are addressing with this concept, and first we have to actually convince decision makers and teachers* (Group 1, P3, male, Slovenia).

### Building confident and competent teachers

Access to and availability of continuous professional development (CPD) opportunities or training associated with PAL within the respective countries appeared to be uncommon and infrequent. Furthermore, among those participants who were delivering CPD to in-service teachers, this was described by one participant as *“trying to fix the problem from the wrong end”* (Group 6, P2 male, Norway). Consequently, to ensure long-term and sustainable changes in practice, the integration of PAL within initial teacher education programmes (ITE) was identified as a priority policy action. Such an investment was felt could substantially influence teacher’s confidence to embrace PAL as a teaching and learning method which teachers could apply to all areas of the curriculum rather than those which are perceived to lend themselves more easily to PAL, such as Maths. Furthermore, participants agreed that integrating PAL in ITE would facilitate the development of Communities of Practice (COP)s as a method of professional learning where teachers could work together to share and reflect on practices and develop their understanding of pedagogical practice relating to PAL. Such was its perceived potential to create change that it was identified as the number one priority for policy and listed as one of the top three priorities of all six workshop groups.

All groups discussed teachers as the gatekeepers to PAL being adopted and used within the classroom. The decision to engage with PAL was felt to be dependent on a teacher’s own values and beliefs about its contribution to their practice. This was not something which participants felt was easy to influence and warned that encouraging teachers to change their practice takes time:

*Teachers often they see themselves as their own boss, so they are very closed around themselves and feel like it’s a loss power if somebody comes in and asks them to change their practices because they feel a lot of pride in who I am, and how I teach, and you can’t come and question that. And that means first of all, it’s really difficult and there’s a big resistance to change*. (Group 4, P5, male, Denmark)

Although participants believed that all teachers had the pedagogical abilities to use PAL in their practice, for those inexperienced with using movement in their teaching, or not physically active themselves, the adoption of PAL was perceived to be even more of a challenge. Therefore, confidence in integrating movement within teaching was felt to be the biggest determinant on whether PAL was used:

*In our new curricula for the primary schools, every teacher can choose goals with movement. It’s nice, but we see the teachers who feel confident in the subject of movement, they do it. If they have a lack of knowledge, they don’t do it. So, it all starts with the teacher they face* (Group 2, P4, female, Belgium).

This was true not only in the short term but also to support continued engagement with PAL. Therefore, participants saw value in supporting teachers by adopting an incremental approach to introducing changes to their practice to balance PAL and more traditional lessons: *“Our first suggestion for the teacher is to choose one small step. One small step is better than nothing”* (Group 5, P5 female, Estonia). The identification and sharing of good and best practice around PAL was frequently highlighted as a priority, to challenge and dispel ambiguity whilst supporting teachers to develop their own understanding of what contributes to effective PAL. Suggestions included both formal (e.g. CPD opportunities such as trainings and seminars) and informal channels (e.g. PAL specific COPs).

### Adopting PAL as part of a whole school approach

To facilitate co-ordinated action and collaboration at a school level, participants identified adopting a whole school approach as a priority action. That is, building a culture within the school to facilitate PAL and aligning it with the school’s development goals. Participants felt that this would give PAL a profile within the school by explicitly acknowledging it as a more engaging way to teach. This was deemed essential to secure commitment from teachers, particularly those who are non-specialist teachers, and secure resource and training to support PAL to address some of their competency needs:

*I think that’s where the whole school approach is quite important. So those people in leadership teams within schools, if they provide support to the teachers and training, hopefully, you could change those attitudes of those teachers* (Group 2, P1, female, UK).

The influence of individual teachers on the adoption and implementation of PAL was frequently mentioned. Participants identified several barriers including a lack of capacity to implement, time to plan and teacher resistance to changing practice. However, a whole school approach was endorsed as a way of helping teachers to introduce sustainable changes in school and avoiding ‘initiative overload’, something that felt particularly important given the impact of COVID-19. Such logistical and capacity challenges were felt to be commonplace when introducing new practices within schools. However, the strategic development of, and commitment to, PAL was believed to foster an in-school culture and organizational norms that support it. For example, one participant referred to the profile and visibility given to movement within a school through the opportunity for teachers to act as role models and using PAL as a way of modelling the importance of being active.

*It is important but it is also important that the teachers understand that I’m not too important in the school and I must move also* (Group 5, P5, female, Estonia).

Many of the enabling factors associated with a whole school approach centred around the creation of a nurturing social environment as part of pupils’ holistic education. This included building quality connections among and between the key stakeholders within the school community. For example, participants felt that teachers would be empowered through opportunities to share and develop reflective practice with colleagues. In addition, it would also demonstrate support and help to legitimize the use of PAL as an accepted pedagogical approach rather than one which only provides more engaging experiences which, as one participant shared, was very important for gaining parental support:

*There’s definitely a big expectation to the schools, from parents, in what they do. And a lot of parents do not see PAL as a proper teaching method. So they will question it.* (Group 4, P5, male, Denmark)

### Strengthening the evidence base

The type of research design was suggested by participants as being important to build the evidence base, with a need to focus on research which is practice-based and applicable to the real word so that teachers can access and directly benefit from it:

*I think we fail terribly. We may be great academics, but we fail to translate that research back to practice in a way that’s digestible so that they [teachers] can then trust and use that*. Group 1, P1, female, UK.

One priority action identified which would facilitate this process and be reciprocally beneficial is using participatory approaches for research, enabling the co-design of research with policy makers and practitioners to ensure that each is informed by and reflective of the other. This would strengthen the utility and impact of the research related to PAL, as one participant reflected:

*I think that’s the future in Denmark, that is that we collaborate also research wise between teacher education and the university because the translation for example, impact on teacher education will go much easier if we do it this way* (Group 3, P3, female, Denmark).

Participants recognised that the evidence-base relating to PAL has grown in recent years but that important gaps still existed, particularly around educational outcomes and effectiveness which could have value when leveraging the interest and support of different stakeholders:

*We don’t have so much strong evidence to say that kids learn the academic content more effectively if they do PAL. So teachers are resistant about it, parents are resistant about it and also policymakers are resistant about it.* (Group 4, P4, female, Portugal)

Other research areas of interest discussed included the holistic or ‘softer’ outcomes of PAL such as social benefits, the transferability of PAL into other cultures and contexts internationally, the differential effects of PAL particularly with different age groups (e.g. secondary students and kindergarten children), capturing pupil and teacher voice and how PAL is experienced by different groups, optimising the implementation of PAL, and the longer term sustainability and effectiveness of PAL. While the method of facilitating this was considered important, of more significance was the inclusion of both practice-based evidence as well as research-informed practice. That is, evidence arising from practitioner expertise and experience, and pupil voice relating to PAL, as well as the use of research evidence on what is effective when designing, implementing, and improving PAL. Furthermore, participants emphasised the need to ensure that different types of evidence were equally valued, accessible, and translated so that teachers felt empowered to develop PAL in their pedagogical practice.

## Discussion

**Fig. 1:**
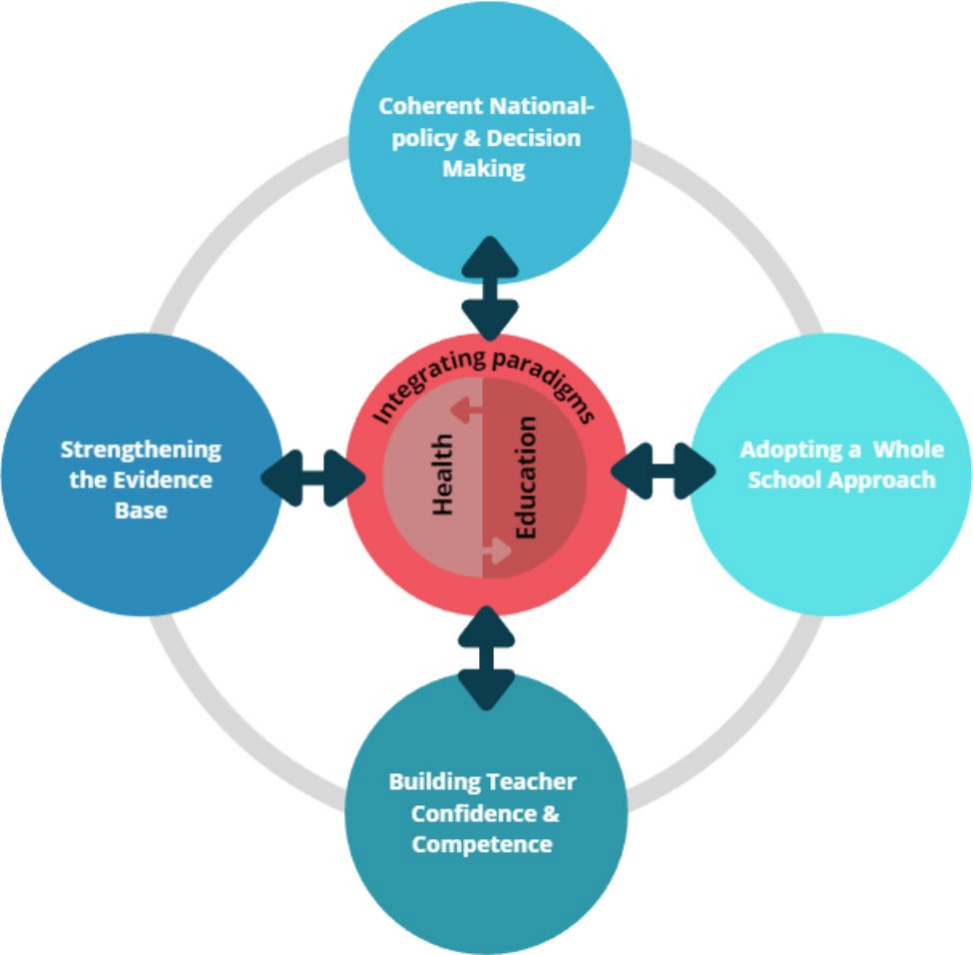
A priority action framework for the development of PAL (movement-centred pedagogy) across Europe

The study advances the understanding of PAL through the co-development of a framework to support PAL adoption and implementation across Europe. Drawing on the expertise of teacher educators, researchers, and policy makers from 13 different countries ensures the broad representativeness of the findings. Advancing the current knowledge base, two novel priorities were identified which focussed on the need for action related to communication and advocacy for PAL and the integration of health and education paradigms. Extending previous work, our findings advance the need to identify gaps and developmental needs in relation to PAL in terms of teacher education [6, 16], strengthening the evidence base [6], influencing policy [6, 7] and adopting a whole school approach [6, 17–19]. The themes are presented as an empirically-informed framework (see Fig. 1) to guide a coordinated set of actions. A summary of the framework is presented in Fig. 1.

Fundamental to the framework is the interrelated nature of the four main priorities and how they directly or indirectly interact with the dominant paradigms of health and education. The arrows within the framework reflect the reciprocity between the different concepts, that is, between the two paradigms of health and education but also the reciprocity between the respective priority and the paradigm integration. Collectively, these represent a combination of top-down and bottom-up approaches which would serve to empower stakeholders at all levels of the system.

A key leverage point which could be exploited is that of reorienting the focus and purpose of PAL, that is the goals and beliefs which align with a collective, complementary, and mutually beneficial agenda. Emphasis should not be given to trying to shift PAL into an educational paradigm or developing an education paradigm which embraces health, but rather one where there is mutual understanding and complementarity of PAL’s meaning and value and the construction of a clear and shared agenda [20]. Such inter and trans disciplinarity would enable to field to move beyond discursive boundaries and disciplinary limitations towards real-world applied research and the continual adaptation of practice [8]. To achieve this, change efforts must influence system architects (i.e. those who can effect change in how the system functions such as politicians and other decision makers such as school inspectorates) and dominant beliefs, with an understanding that the mechanisms by which this is achieved will vary greatly due to contextual and cultural sensitivities [21].

To influence system architects, there is a need to advance the knowledge base surrounding PAL. To date, the evidence for PAL has been driven by a preventative public health perspective and contemporary societal issues of sedentarism and physical inactivity [22]. While research has progressed beyond studies of effectiveness to understand teacher behaviour and PAL implementation, little work has taken place integrating this work within the educational field such as how PAL can influence teachers’ perceptions of pupils’ knowledge and learning, in ways that reflect the broader purposes of education. Encouraging signs of PAL as an interdisciplinary paradigm are emerging within education-oriented research around teachers’ pedagogical considerations about PAL and why it is used in education [23, 24]. However, to embed PAL within policy, school systems and teacher practice, an integrated understanding, combining health and education paradigms, associated theories and methodologies is required to progress the field. For example, theoretical reciprocity of the disciplines has oriented knowledge production toward pedagogy and didaktikks and a more layered understanding of teachers’ perceptions of and experiences with PAL [25].

Some progressive countries (e.g. Norway, and Finland) lead the way with PAL, aligning policy and decision making around health and education [7, 26] ). Improved advocacy, drawing on an integrated research and practice agenda can support others to do the same. Resultant policies, co-developed with all system stakeholders, should prioritise complementary health and education goals, placing holistic development at the heart of the educational experience. The impact of such an approach is evidenced in recent research comparing PAL across three European countries [7]. Activating PAL through a whole-school approach is an essential system goal. To achieve this, it is fundamental to build competent and confident teachers through high-quality ITE. Examples of short-term and longer-term professional development training opportunities specific to PAL have been documented which have demonstrated positive effects on teacher readiness and skills to integrate movement into teaching [16, 27]. However, the inclusion of PAL content in ITE is essential to ensure early exposure to PAL and promote competency development. The recent cross-European PAL teacher training curriculum provides a blueprint to achieve this goal [28] which includes evidence-based information and a behaviour change approach to develop the capability of teachers, increase their motivation for PAL and capaitalize on opportunities available for PAL delivery within an educational setting.

### Implications of this study

The results and subsequent discussion paint a strong argument to reframe PAL using terminology favourable to both paradigms. While “PAL” is widely accepted within health, the challenge for broader uptake and use beyond early adopters prevails. The phrase “physically active” has strong alignment with a health paradigm, perhaps creating friction to the broader uptake by educationalists. Reframing terminology can increase accessibility for all stakeholders, promoting an integrated understanding. Following extensive discussion, the author team would like to propose “movement-centred pedagogy” as a term that integrates the core of both paradigms. *Movement* progresses beyond the narrow vision of minutes of moderate to vigorous physical activity often associated with PAL, reflecting a broader appreciation of physical activity aligned with recent definitions [29] and messaging from the World Health Organization [30]. *Centered* reflects the purpose of the movement and its role in providing a connection with the subject content. Rather than predefined educational outcomes, *pedagogy* reflects a wider conception of the teaching-learning process in education that includes teachers’ choices and judgements about how, why, where and when to integrate movement in teaching. The arguments presented here to rename PAL “movement-centered pedagogy” are similar to those presented in earlier work, which argues for using the term “movement integration” in place of classroom physical activity [1]. However, our proposition provides a more inclusive and holistic perspective, which embraces the educational discipline’s language, and places a spotlight on the purpose and function of movement and how it is enacted. In doing so it provides a more nuanced understanding and greater conceptual clarity.

### Strengths and limitations

This is the first study to co-produce priority areas for action to support the adoption and implementation of PAL across Europe. It extends previous work conducted in this area by drawing on the experiences of multi-stakeholders from different cultural contexts and education systems across countries in Europe, especially those that have been at the forefront of implementing PAL on a national scale (e.g. Norway and Denmark).

The convenience sampling of participants and modest sample size may result in some bias, for example, a low number of teachers or teacher educators representing practitioners, however the sample included multi-stakeholders from a variety of different roles. Furthermore, many participants also held a teaching qualification and collectively provided a depth of understanding and information richness with regards to the adoption and implementation of PAL [31]. Although participants warned against a one size fits all approach, there appears to be shared learning which is transferable into other country contexts across Europe, and likely more widely internationally. The replication of this research with stakeholders from countries, including low and middle income, across different regions (e.g. the Americas and Western Pacific) would be an interesting point for exploration in further research. Furthermore, while the framework provides a basis for action, it does not provide guidance on how to implement these actions. The identification of such next steps would similarly benefit from additional multi-stakeholder collaborations and consensus on their translation into policy, practice and research [32].

## Conclusion

The priority action framework facilitates PAL adoption and implementation across Europe, identifying five key areas for action. Central to the success of border PAL dissemination is the integration of the health and education paradigms. This is essential to enact change through collective, complementary and mutually beneficial agendas. To achieve this aim, reframing PAL as movement-centred pedagogy would provide a more holistic and inclusive perspective to mobilise and galvanise action across multiple sectors with a range of stakeholders.

### Electronic supplementary material

Below is the link to the electronic supplementary material.

Supplementary Material 1 COREQ Checklist

## Data Availability

The datasets created and analyzed during the present study are available from the corresponding author upon reasonable request.

## Footnotes

1 ACTivate (Activate Classroom Teachers) is an Erasmus funded Strategic Partnerships in Higher Education project (2019–2022), six-nation partnership (Western Norway University of Applied Sciences (Norway), Leeds Beckett University (UK), LIKES (Finland), The Mulier Institute (The Netherlands), University College Denmark, (Denmark) and University of Porto, (Portugal)). The main objective is to co-create, with teachers and other school stakeholders, an innovative European-wide open access PAL education programme, a PAL curriculum, a PAL web portal and a community of practice (www.activateyourclass.eu).

## Abbreviations

PAL: Physically Active Learning
ITE: Initial Teacher Education
PE: Physical Education CPD:Continuous Professional Development
COP: Communities of Practice
